# Evaluating the Link between Visual Attention Bias and Emotion Dysregulation of Young Children

**DOI:** 10.1007/s11126-024-10089-4

**Published:** 2024-08-28

**Authors:** Febe Brice, Christa Lam-Cassettari, Brigitte Gerstl, Valsamma Eapen, Ping-I. Lin

**Affiliations:** 1 Discipline of Psychiatry and Mental Health, School of Clinical Medicine, University of New South Wales, New South Wales, Australia; 2 Academic Unit of Child and Adolescent Psychiatry, South Western Sydney Local Health District, Sydney, Australia; 3 Department of Mental Health, School of Medicine, Western Sydney University, Sydney, Australia; 4 Department of Psychiatry and Behavioral Neuroscience, Saint Louis University, Saint Louis, USA

**Keywords:** Attention, Emotion dysregulation, Temper tantrum, Pre-school children, Eye gaze

## Abstract

The ability to regulate emotions is vital to successful social interactions. This study explores whether visual attention bias is associated with emotion dysregulation (ED) in early childhood. Parental reports of child ED (Child Behaviour Checklist (CBCL) and Temper Tantrum Scale) were examined in relation to child visual attention bias whilst viewing emotional faces. Results indicated that the level of eye gaze fixation towards emotional images and faces was associated with ED when social function (measured with the Social Responsiveness Scale), gender, age, and attention problems (measured from the CBCL subscale), were adjusted. The modifying effect on visual attention bias was evaluated using interaction analysis in the generalized linear model. The level of visual attention bias, indicated by the proportion of eye gaze fixation time on areas of interest (AOIs) in images displaying unpleasant emotions (such as anger), was inversely associated with the level of externalising problem behaviours (*p* = .014). Additionally, the association of eye gaze fixation time for AOIs displaying negative emotional cues with the level of externalising problem behaviours varied by age (*p* = .04), with younger children (aged < 70 months) demonstrating a stronger association than older children (aged $\ge$ 70 months). Findings suggest that young children with greater ED symptoms look less at unpleasant emotional cues. However, this relationship is attenuated as children become older. Further research to identify objective biomarkers that incorporate eye-tracking tasks may support prediction of ED-related mental health issues in the early years.

**Supplementary Information:**

The online version contains supplementary material available at 10.1007/s11126-024-10089-4.

## Introduction

Emotion regulation involves an individual's ability to self-regulate, controlling or employing coping mechanisms to effectively manage emotions in response to stimuli. Effective emotion regulation allows individuals to adapt to and navigate their environment, maintain their emotional well-being and engage appropriately in social interactions [1, 2]. Emotion dysregulation (ED) is the inability to regulate one’s own emotional responses in a manner that is considered appropriate for the social context, thus resulting in poor emotional experiences including frequent mood swings, emotional outbursts, irritability or acts of aggression. ED is highly prevalent, present in 3% to 20% of children and youth in general [3], and found in 13.9% of the general adult population [4]; illustrating its impacts can be severe and far-reaching even into adulthood.

ED early in life can be an indicator of poorer social function and behavioural problems. Previous research has shown that preschool children who cannot self-regulate their emotions demonstrate externalising behaviours and reduced ability to socialise appropriately, which can present as aggressive behaviour, irritable mood or tantrums [2]. Early behavioural problems such as aggressive behaviours towards themselves or others, not only impact a child’s ability to learn and develop their interpersonal skills but are also known precursors for physical and mental health issues later in life [5, 6]. Longitudinal studies have demonstrated that aggression in school-aged children increases the risk of future violence in adolescence through to adulthood [5–7]. Similarly, ED has been shown to contribute to multiple psychological issues, including, but not limited to mood disorders, sleeping disorders, psychological traumas, personality disorders, and aggressive tendencies or behavioural disorders such as oppositional defiant disorder or conduct disorder [8, 9].

ED is also a transdiagnostic trait in developmental disorders. Meaning, it not only appears across multiple disorders, but also modulates the shared pathological components across these diagnoses, such as genetic factors, further contributing to their maintenance [10, 11]. Specific examples where ED may be a prominent trait include autism spectrum disorder (ASD) and attention deficit hyperactivity disorder (ADHD) [12, 13]. Studies have demonstrated that ED is more likely to be present in children with a diagnosis of ADHD and ASD, compared with neurotypical children [14, 15]. Not only is ED more prevalent in neurodevelopmental disorders, but it can play a role in the development and maintenance of these [9, 11, 16]. Studies have found that ED is closely related to the core features of ASD, with the features of aberrant social development and repetitive behaviours particularly making them more vulnerable to ED [17, 18]. The exact relationship between ADHD and ED is currently debated, but there is also evidence suggesting ED directly accounts for ADHD-related impairments, beyond symptoms of inattention and hyperactivity-impulsivity, rather than just being a secondary result of these deficits [19, 20]. Furthermore, there is debate as to whether ED is a core feature of ADHD, or if it is predominantly only a feature of the hyperactivity-impulsivity subset [21]. Regardless, studies have shown that ED in ADHD is a predictor of more severe symptoms in childhood, as well as poorer long-term clinical and educational outcomes later in adulthood [21, 22]. With ED being not only a risk factor for behavioural issues and psychopathologies later in life, but a potential exacerbator of certain disorders; it is evident that learning emotion regulation skills, in early childhood, is critical in developing and maintaining healthy psychological wellbeing later in life [2, 23].

To minimise the impact of ED, early detection and appropriate interventions are essential. Untreated ED and its related behavioural and psychological consequences can cause continuing problems, impacting not only a child’s cognitive and emotional development but also the wellbeing of their family. Preschool years are a key stage for developing positive or maladaptive emotion regulation strategies. For children and their families to achieve the best outcomes, it is important to determine if an intervention is required and engage in early interventions before complexities and comorbidities develop [24].

Detecting early childhood ED to implement early interventions is simple in theory; however, putting this into practice effectively is more challenging. With young children, for example, it is often difficult to determine whether their symptoms are a result of ED, part of normal child development, or due to an associated developmental disorder. Hesitation in diagnosing a child with ED to determine if it is a phase using a “wait and see” approach consequently delays the implementation of early intervention considered more efficacious [25]. In the context of detecting ED early in children, easy-to-use screening methods suitable for use at community level could support early detection, expedite access to interventions, and support parents, educators and healthcare providers to address ED effectively [26].

Eye-tracking, a potentially accessible tool, may serve as a simple screening method for ED in young children. It employs various indices to explore the connection between visual emotional processing and ED. This method allows continuous monitoring of attention deployment to emotional stimuli, facilitating the examination of the relationship between attentional processing and mental states. [27]. Other studies use pupil size, initial fixation time, initial fixation location, fixation count and fixation duration to explore how emotional and/or mood disorders impact visual attention to emotional expressions [28–30].

Visual attention, the cognitive ability to select or filter information from a visual stimulus [31], shapes the way humans learn, self-regulate and behave [32]. Visual attention is driven by the balance between preference and aversion towards stimuli, which is more likely to reflect emotional responses to the environment than verbal expressions that can be confounded by social desirability. There is evidence that visual attention plays a key role in emotion regulation, with correlations observed between visual attention and presentations associated with ED, such as aggressive behaviour, irritability or neuroticism [33, 34]. Visual attention bias refers to the variation in attention towards an object reflected by eye gaze fixation patterns. Eye-tracking technology is frequently utilised in studies to measure visual attention bias and examine its role in various psychological and emotional disorders. Various methods can be used to measure visual attention bias, including but not limited to dot-probe, spatial cueing, visual search with irrelevant distractor and attentional blink tasks. Although the attentional blink task is highly sensitive at measuring when participants would preferentially attend to an emotional stimulus [35], the notably high individual variation in responses to this task may not necessarily be attributable to emotion regulation [36]. There is strong evidence that eye-tracking technology can sensitively measure visual attention bias, and provide insight into the attentional processing of emotional stimuli thereby acting as a potential biomarker for ED. However, there is limited literature in this area regarding young children, and whether the same methods can be utilised to detect ED in them.

It has been proposed that aggressive children would be more sensitive to cues of hostility than their less aggressive counterparts, even going as far as to misinterpret a cue as being more hostile [33, 37]. An eye-tracking study examined the visual scan paths of 30 adults when they gazed at emotional expressions, to determine whether there was a correlation between neuroticism, another trait associated with ED, and visual attention bias. Individuals were drawn to traits congruent with their own, in which those with higher levels of neuroticism attended more to the eye region of fearful faces. [34]. A study with a smaller sample size demonstrated a similar trend where aggressive adolescents when shown still photographs of cartoon scenes, became fixated on the scenario with traits congruent to their own (e.g., hostile cues) [38].

However, other results are contradictory. For example, Horsley et al. [33], examined the eye movements of 60 children aged 10–13 years and found that children with higher levels of aggression fixated more on the non-hostile cues than their less aggressive counterparts [33]. Similarly, a study involving 30 adults also showed an inverse relationship between eye gaze duration towards hostile pictures/videos/still-images and level of aggression. When participants were shown a hostile character, individuals who had exhibited more aggressive tendencies became fixated on non-facial areas while less aggressive individuals focused on areas in the facial region such as the eyes [39]. Thus, both studies indicate that individuals with traits of aggression showed aversion to scenarios involving hostility.

Other research examining ED and attention bias have reported trends where individuals with higher levels of ED showed aversion to emotional stimuli. A study examining attention bias and the behavioural inhibition temperament type in 12 children aged 5 to 7 years found that those with higher levels of behavioural inhibition exhibited fewer gaze shifts towards a stranger in a live social interaction [32]. An evaluation of aggression and attention bias in 76 juvenile delinquents showed that individuals with antisocial tendencies demonstrated an aversion to hostile stimuli [40]. Both studies proposed that avoidance of the emotional stimuli was an involuntary emotion regulation strategy to reduce emotional arousal and regulate negative emotions [32, 40].

While multiple studies have been conducted to explore the role of visual attention bias in ED, most of these were conducted with either adult participants or older children. As discussed previously, the development of emotion regulation skills early in life is essential to the wellbeing of individuals later in life [2]; as a result, early detection is necessary in order to deploy early interventions targeting ED. It is unclear whether eye-tracking platforms would be a feasible screening tool for ED in young children, as it is difficult to extrapolate from the existing literature on older children and adults to the younger age group. Additionally, many of the studies utilised only one self-reporting tool to measure traits of ED [32, 34, 38–40] thus increasing the risk of reporting biases. These factors, compounded with the small sample sizes featured in many of these studies, can confound the relationship between visual attention bias and ED. Furthermore, the generalisability of results from several of these studies is limited due to the specialised nature of the sample.

Uncertainty persists regarding the predictive capacity of visual attention bias for ED and related behaviors in children. This project seeks to determine the role of visual attention bias in the development of ED in young children. The study aims to investigate whether visual attention, indicated by gaze fixation duration for specific areas of interest, correlates with the severity of parent-reported measures of ED. Our hypothesis posits a correlation between visual attention and ED severity in children, with a higher proportion of gaze fixation time towards areas with hostile cues (e.g., the eyes of an angry face) in children exhibiting higher levels of ED. Additionally, we aim to explore whether this relationship is influenced by social function (as reported by parents) or other individual attributes, such as age and sex.

## Methods

### Conceptual Framework

**Fig. 1:**
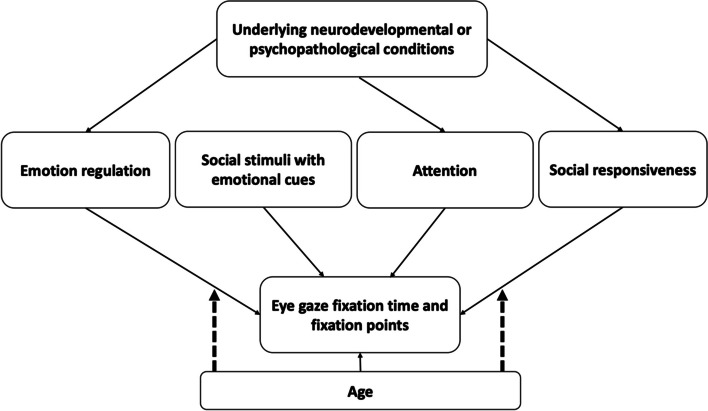
The relationship between the key variables illustrating how the main and interaction effects were examined. The dashed arrow indicates the modifying effect, and the solid arrow indicates the direct effect

The study design is based on the conceptual framework, which proposes that a child's processing of social information, as reflected in visual attention, is influenced by factors such as ED, social responsiveness, and the ability to sustain attention to an object (see Fig. 1). Specifically, social function was adjusted in the model due to the role of social information processing in emotion regulation-related behavioural responses as well as visual attention to social cues [41]. Further, this framework suggests that gender and age may not only influence visual attention, but also potentially modify the relationship between ED and visual attention. Evaluating the modifying effect of age could be used to explore heterogeneity in the link between ED and visual attention.

### Participants

Children aged 3–8 years were recruited for this study via personal networks and social media in Australia. Participants were excluded if they: 1) had a major visual impairment, 2) did not have access to a laptop with a reliable internet connection and webcam, or 3) if their parents were not proficient in English. The minimum age of three was chosen as the eye-tracking measure required that participants minimise movement and remain seated and gaze at a computer screen for at least two minutes. A total of 50 children consented to the study. Demographic data was collected and managed using Research Electronic Data Capture (REDCap) software hosted by the University of New South Wales [42, 43].

Descriptive statistics of participant demographics and scores from ED and social function measures are described in Table 1. The sample (n = 50) was predominantly male (n = 31, 62%), with most children being born in Australia (n = 35, 70%). Aside from premature deliveries (n = 6, 12%) few reported birth complications (n = 3, 6%).

**Table 1:** Characteristics of the study participants (total N = 50)

Variables		N (%) or M (SD)
Sex		
Female		19 (38)
Male		31 (62)
Age (months)		70.32 (21.28)
Birthweight (kg)		3.35 **(**0.47)
Premature births		6 (12)
Labour complications		3 (6)
Country of birth		
Australia		35 (70)
USA		10 (20)
Other		5 (10)
Ethnicity		
African American		14 (28)
Asian		10 (20)
Caucasian		9 (18)
Other		6 (12)
Unspecified		11 (22)
Language spoken at home		
English		38 (76)
Mandarin		4 (8)
Multiple		3 (6)
Other		5 (10)
Parental education		
Year 10		1 (2)
Year 12		0 (0)
Undergraduate degree		18 (36)
Postgraduate degree		24 (48)
Other post-school qualification		7 (14)
CBCL T-scores		
Total problems		60.72 15.86
Internalising problems		61.28 16.06
Externalising problems		55.26 10.76
Attention problems		57.32 8.42
SRS-2 total T-score		61.94 15.36
TTS total score		21.46 9.71

*M* mean, *SD* standard deviation, *CBCL* Child Behaviour Checklist, *SRS* Social Responsiveness Scale, *TTS* Temper Tantrum Scale

Ethics approval was received from the Institutional Human Research Ethics Committee of the University of New South Wales, Sydney (Ethics approval number: HC210989). The parents/caregivers of the participants were informed about the purpose and procedures of the study and gave written informed consent before participating.

### Measures

#### The Level of ED

The level of ED in children was measured using the Child Behaviour Checklist (CBCL) [44] and the Temper Tantrum Scale (TTS) [45]. CBCL is a parent-report measure used to assess children's behavioural problems. The CBCL’s total, externalising, internalising and attention problems subscales were used for this study. Both the Early Years (ages 1.5–5 years, 99 items) and the School Age (ages 6–18 years, 113 items) versions were used to cover the age range of 3–8 years. The CBCL provides t-scores and a percentile for comparison with a normative sample of the same age group, indicating whether they are within the normal range, borderline clinical range or the clinical range [44]. The TTS is a 10-item parent-report questionnaire that measures loss of temper in children, a behavioural trait common in ED. While temper tantrums are indeed common in childhood and not always abnormal, this scale assesses their frequency and related behaviours to determine if they are clinically concerning [45].

#### Social Responsiveness

The Social Responsiveness Scale (SRS-2) [46] is a 65-item questionnaire that measures social awareness, cognition, communication, motivation and the presence of restricted interests or repetitive behaviours. To cover the age range included in the study, both the Pre-School (children aged 2.5–4.5 years) and the School Age (children aged 4–18 years) forms were used. The SRS-2 has high internal consistency and is a reliable measure of social impairment [46].

#### Eye-tracking Test

The eye-tracking test was undertaken by participants using a weblink to log into the cloud-based webcam eye-tracking service platform RealEye [47] to observe a series of visual images preloaded into a test (Appendix S1). Participants completed a calibration phase in which they gazed at dots on the screen before proceeding to the test phase. The test phase comprised of 5 images, that were a mixture of cartoon and real-life media. Two images consisted of singular human faces (displaying either happy or angry emotional expressions) staring at the viewer, and three images showed an interaction between two characters, in which the viewer acts as a bystander without the possibility of eye-contact with the characters in the image. 

Fixation refers to a cluster of eye gaze points that are physically and temporally close to each other. To assess fixation, areas of interest (AOIs) for each image had to be determined. For images with a single face, the AOIs selected were the main object of social interest (e.g., the eye and mouth regions). For images with multiple characters, the AOIs selected were the whole faces, to compare visual attention towards characters with different emotional cues (e.g., happy versus angry). For this purpose, parameters including minimum fixation duration, noise reduction, and gaze velocity threshold were automatically calculated by the RealEye software algorithm. This platform has been used in multiple studies with an accuracy of approximately 100px (~ 1.5 cm) with an average error on the visual angle of ~ 4.17 deg [47].

Differences in eye gaze fixation time in specific AOIs (e.g., eye regions of the human face) indicates visual attention bias. In our eye-tracking study, we selected the eyes as primary AOIs to investigate visual attention and emotion regulation in response to hostility. The eyes convey substantial emotional information, serving as key indicators of emotional states and intentions. Additionally, the eyes' quick reflection of emotional state changes aids in capturing real-time responses to hostility, enhancing our understanding of the interplay between visual attention and emotion regulation. By analysing both fixation duration and the proportion of fixation time for the images with two AOI, competing interests can be adjusted for and the confounding effect of the total attention span for the whole image. Focussing on the visual attention towards objects in the social context (e.g., a picture of a child being laughed at by another child) could provide insight into how emotion can be regulated through social information processing.

### Statistical Analysis

Statistical analysis was performed with both JMP Pro 16 [48] and STATA 17 statistical software [49]. Descriptive statistics were presented as frequencies and proportions for categorical variables, and as means and standard deviations (SD) for continuous variables. Pearson correlations were run to determine correlations between the ED scores obtained by participants and visual fixation time on AOI in the stimuli. Generalised linear models were conducted, with models being adjusted for the child’s sex (male versus female), the child’s age (age < 70 months versus > 70 months), and the SRS-2 score. The multi-variable generalised linear model to evaluate the interaction effect can be expressed as: *visual attention bias* = *β0* + β1*ED + β2*age + β3*age*ED + β4*gender + β5*SRS + β6*attention problem. A stepwise regression model to re-analyse the data to confirm the most influential predictors for visual attention (i.e., eye gaze fixation time).

The data will be made accessible to researchers with proper ethics approval.

## Results

**Fig. 2:**
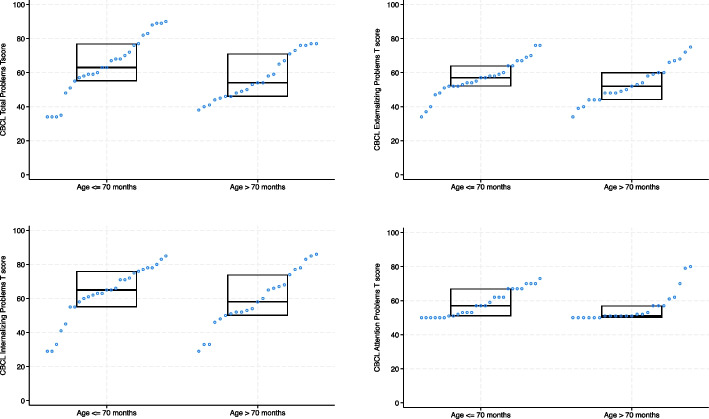
The distribution of ED-related variables and social responsiveness stratified by age

Figure 2 displays box plots of the scores achieved by participants in each of the measures. The mean T-score for each measure was moderately high (except for the CBCL externalising and attention problem T-scores) where values above 60 indicate a potential problem.

### ED, Social Function and Visual Attention Bias

To explore whether ED was related to visual attention bias, the Pearson correlation test was conducted between the measures of ED (TTS total, CBCL total, externalising, and internalising problems) and the gaze fixation times for each AOI. To explore whether there was a correlation between social function and visual attention bias, Pearson correlations were also conducted for the SRS-2. The correlation coefficients are displayed in Table 2, where negative values indicate a negative correlation between the questionnaire score and gaze fixation time on the stimuli.

**Table 2:** Pearson’s correlation coefficients for AOIs and scores from measures of ED and social function

Area of interest (AOI)	CBCL total problems T-score	CBCL externalising problems T-score	CBCL internalising problems T-score	SRS-2 total T-score	TTS total score
1	-0.1231	0.0507	-0.0910	-0.1054	0.0069
2	0.1667	0.1746	0.0128	0.1001	0.2362
3	-0.3770**	-0.3338*	-0.2854*	-0.2405	-0.2004
4	-0.3494*	-0.3864**	-0.3336*	-0.2889*	-0.0993
5	-0.1578	0.0334	0.1614	-0.2362	-0.0691
6	-0.3681**	-0.2185	-0.0615	-0.4203**	-0.3760**
7	0.1582	0.1686	0.0739	0.0823	0.1025
8	-0.1023	-0.0005	-0.0629	-0.1955	-0.0573
9	-0.1806	-0.0411	-0.0568	-0.1900	-0.1567
10	-0.2636	-0.1012	-0.0289	-0.2489	-0.2291
11	-0.0594	0.1046	-0.0137	-0.1930	-0.0363
12	0.0044	0.1392	-0.0411	-0.2259	0.0409
13	-0.0911	-0.1542	-0.1038	-0.2438	-0.2362
14	-0.1422	-0.2566	-0.0935	-0.2752	-0.2893*
15	-0.1816	0.1389	-0.0072	-0.3252*	-0.1538
16	-0.1580	0.2134	0.1052	-0.2351	-0.0496
17	0.0270	0.0126	0.0387	-0.0974	-0.0502
18	-0.0764	-0.1267	-0.0678	-0.2022	-0.1228
19	-0.1800	-0.0794	-0.0238	-0.0898	-0.1432
20	-0.1341	0.1578	0.0763	-0.2401	0.0200
21	-0.3576*	-0.2277	-0.1822	-0.3056*	-0.2479
22	-0.2014	-0.2419	-0.2365	0.0092	-0.2473
23	-0.1791	-0.2085	-0.1803	0.0511	-0.1978
24	0.0913	0.0212	-0.0826	0.1388	0.1565
25	0.0014	0.0465	0.0125	-0.0352	0.0899
26	-0.2314	-0.0285	0.0140	-0.2457	-0.3567*
27	-0.2965*	-0.0138	-0.0887	-0.2862*	-0.2558
28	-0.2447	-0.1347	-0.0650	-0.3028*	-0.3218*

Significant correlations are in bold

*p < .05. **p < .01

**Fig. 3:**
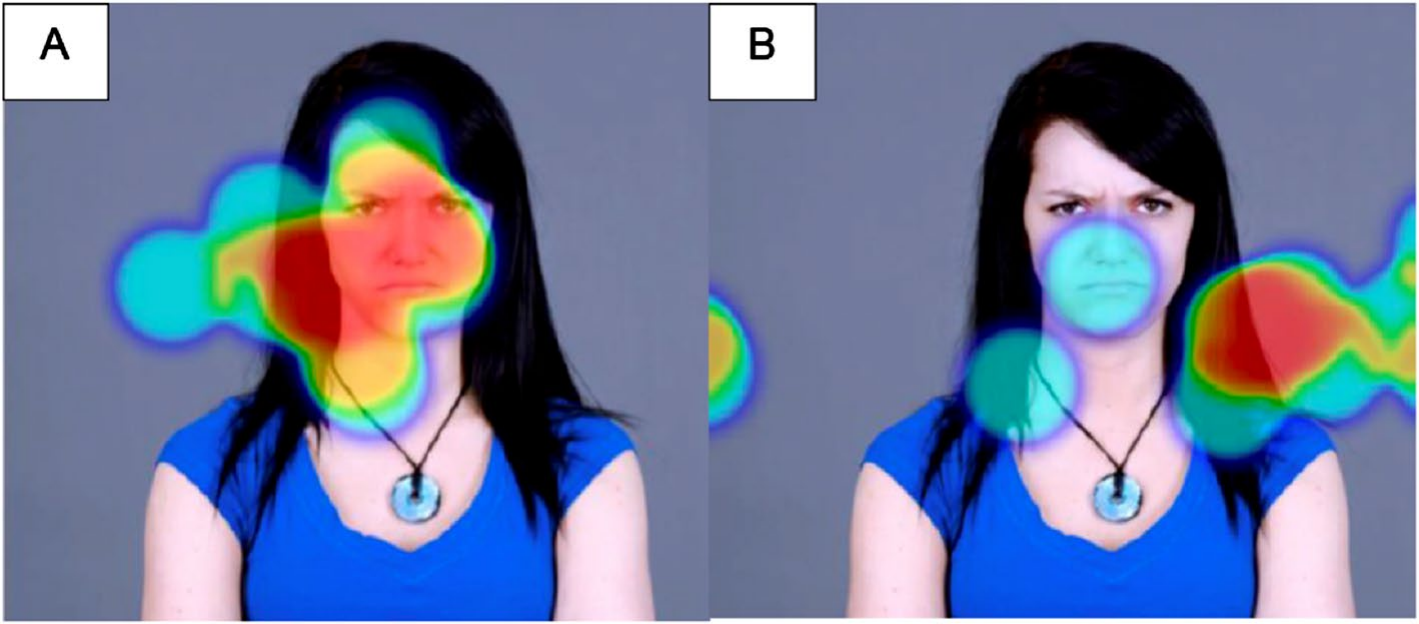
The heat map of eye gaze fixation time in AOI 3 (the eye region of an angry face): (A) the individual with a lower level of CBCL externalising problems; (B) the individual with a higher level of CBCL externalising problems

**Fig. 4:**
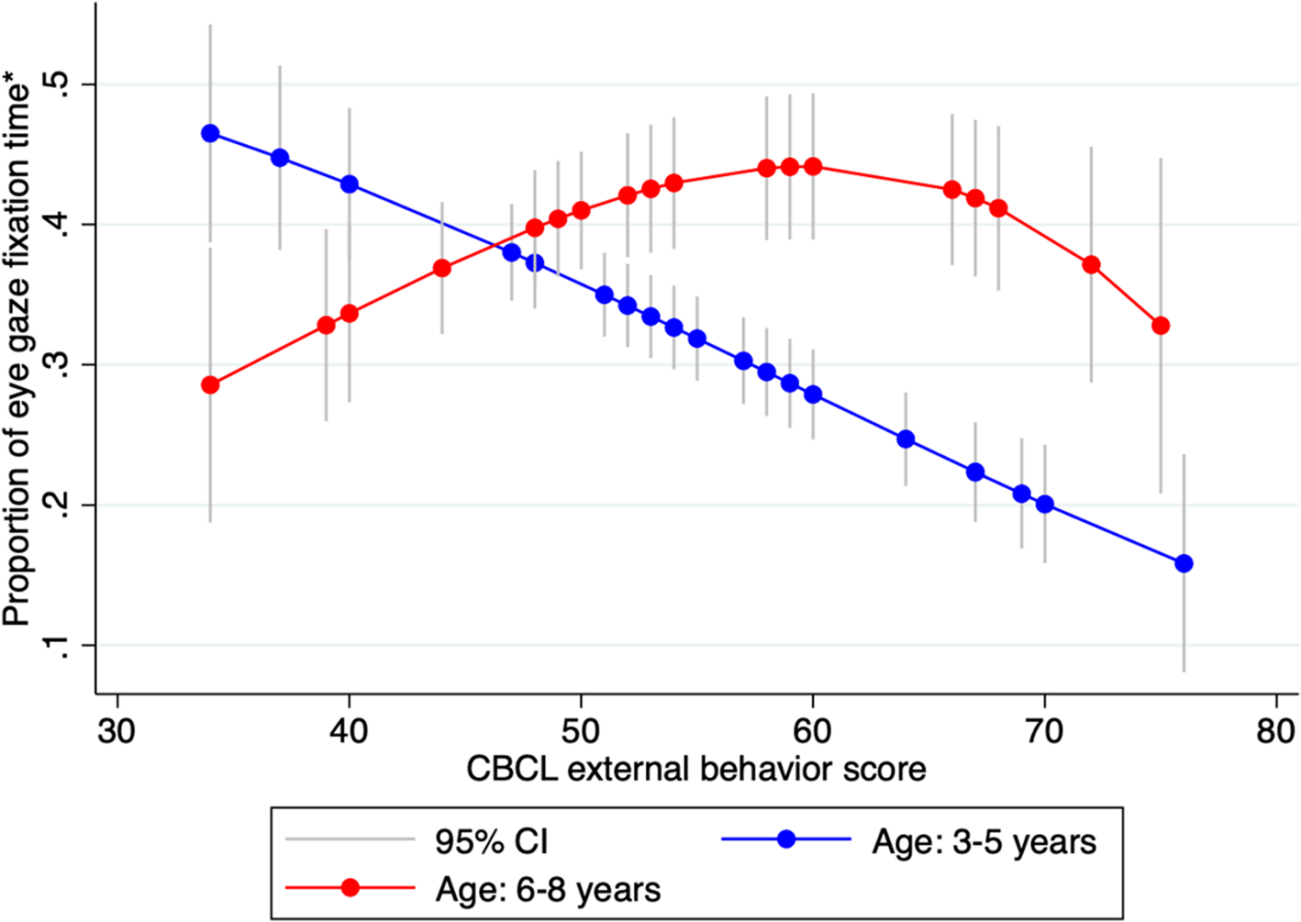
The relationship between the externalising problem score and visual attention bias in two age groups (< 70 months and $\ge$ 70 months, respectively)

The AOIs and ED measures that showed significant correlations (seen bolded in Table 2) were analysed with the multi-variable general linear model, controlling for age (< 70 months versus > 70 months), sex (male versus female) and social function (SRS-2 score). There were no significant associations between any of the AOIs and the TTS total score. However, AOI 3 (angry face) and AOI 4 (happy face) showed significant associations with other measures. Significantly inverse associations were found between the CBCL externalising problems and AOIs 3 (*p* = 0.001) and 4 (*p* = 0.002). Figure 3 depicts AOI 3 (the eye region of an angry face) with eye gaze fixation heatmaps demonstrating this trend. Notably, age was associated with CBCL externalising problems and AOI 3 (the angry face) (*p* = 0.04). Since AOI 3 and AOI 4 were in the same picture frame, visual attention bias could be driven by competing visual stimuli. Therefore, we used the proportion of eye gaze fixation time within AOI 3 in total time over the two AOIs as an indicator for visual attention bias. A graph depicting this relationship is shown in Fig. 4, where there is an inverse correlation between the externalising problem scores of children younger than 70 months and the proportion of fixation time on AOI 3 in the total fixation time on both AOIs in the same image, whilst children older than 70 months demonstrated a positive correlation. Further analyses were conducted to examine the association between the number of fixation points and behavioural outcomes, which yielded similar results. The key variables examined in the study and their relationships can be seen in Fig. 1.

## Discussion

The current findings suggest that visual attention bias in response to images could be associated with ED manifested as externalising behaviours. Specifically, children with higher levels of externalising behaviours might exhibit less visual attention towards images with unpleasant emotions, such as anger, than children with lower levels of externalising behaviours. Notably, the association between visual attention and ED expressed as externalising behaviours seems to be more prominent in younger children than older children. Interestingly, although the association between internalising problems and visual attention bias was at best marginal (since it was not significant after adjusting for other covariates), children with higher levels of internalising problems seemed to exhibit less visual attention to positive stimuli (such as a happy face). These findings may shed novel insight into cognitive mechanisms underlying emotion regulation in children.

Theories surrounding the role of visual attention bias in ED, particularly in young children, are mixed. The social information processing model proposed by Crick and Dodge (1994) suggests that aggressive individuals are more likely to interpret and respond to hostile stimuli [37]. Another model suggested by Gross (2013), describes attention deployment as a strategy used to downregulate emotions [50]. The findings from the present study are more consistently explained by the latter theory. It appears that children who have higher levels of ED are more likely to experience greater challenges in regulating their emotions in response to negative stimuli and hence are more likely to utilize attention deployment to regulate their emotions by visually avoiding the source of discomfort [51]. This trend is demonstrated in our study, where higher scores on the CBCL’s total problems and externalising problems, indicating more behavioural issues and higher levels of aggression and irritability, resulted in a visual attention bias away from the negative stimuli. This is consistent with the findings of Horsley et al. (2010), which observed in a study of 60 children, aged 10–13, that more aggressive individuals were more likely to fixate on non-hostile cues than their less aggressive counterparts [33]. Another study examining attention biases towards emotional faces in young boys also found that those with increased childhood adversity levels had an attention bias away from negative emotional stimuli; thus further illustrating visual avoidance as an emotion regulation strategy [52].

Note that externalising problems refers to behaviours such as aggression and temper loss, whereas internalising problems consist of anxious or depressive symptoms [53]. It is possible that the visual attention characteristics may be different in externalising versus internalising behaviours. In this regard, it has been proposed that anxious and depressed individuals attend more to negative information, further maintaining their depressive mood and symptoms [54]. This potentially explains the visual aversion to positive stimuli seen in children who scored high on the internalising problems scale. A study examining adolescents with anxiety elicited a similar trend, in which participants visually attended to negative faces rather than positive faces [55].

The present results do not concur with the social information processing model [37]. Despite evidence that there is a significant correlation between aggression and attention towards angry faces, we believe that the discrepant findings observed in our study compared to previous studies is likely due to differences in methodologies [38, 56]. In this regard, several variables including age, type of eye-tracking task, stimuli and differing self-report measures, are all possible confounders of the relationship between visual attention bias and ED.

Interestingly, the studies that appear to have incongruent results to our own are based on older samples. Our findings indicate that the inverse correlation between externalising problem behaviours and attention bias for negative stimuli was weakened in the older participants (aged > 70 months). Prior research suggests visual attention patterns when engaging with emotional stimuli can develop and change with age. Typically developing preschoolers showed an increased attentional bias for emotional versus neutral facial expressions during a free viewing task from 2.5 to 5 years of age [57]. One study investigating age-related differences in the viewing of emotional faces in children and adults found that adults and older children aged 12 years, consistently directed their visual attention to the face rather than the contextual information of a scene. On the contrary, younger children were found to allocate their attention between the face and the contextual information, particularly when trying to make an emotional judgment. Such results indicate developmental changes in visual attention when perceiving emotional stimuli, in which, older participants are more likely to attend to an emotionally negative face than a younger participant [58]. Our findings, taken in conjunction with the current literature, leads us to believe that attention deployment as an emotion regulation strategy is attenuated with age.

The present study demonstrated a significant inverse correlation between ED severity and visual attention for emotionally unpleasant stimuli in young children. Attentional deployment is widely regarded as a positive strategy of emotion regulation, often the target of therapies to help regulate negative moods. However, in some cases, it can be a maladaptive strategy [51]. For instance, it is well-known that excessive avoidant behaviour is a key feature of anxiety disorders and is often associated with psycho-social impairment [59]. The avoidant gaze pattern we see in our study amongst more irritable young children when looking at negative stimuli could be considered as a biomarker for maladaptive attentional deployment.

Determining this correlation between gaze pattern and ED, as well as the influence of age on this relationship, has significant implications for the screening and early detection of ED. While further research is needed, determining if an avoidant gaze pattern towards negative stimuli is indeed a biomarker for ED could provide an objective measure of screening for ED, thus potentially facilitating early detection. Furthermore, if this avoidant gaze in children with ED is deemed a maladaptive emotion regulation strategy, this could inform new intervention strategies. While attention deployment is already the target of some therapies for depression or aggression in adults, the goal is usually to encourage consumers to use visual avoidance to down regulate negative emotions [51, 60]. We believe the opposite, where visual attention to an object of discomfort is encouraged, could also be employed as a therapy to counteract the maladaptive avoidance signifying ED in young children.

This study has a variety of methodological strengths. Firstly, the use of eye-tracking software and a mixture of stimuli containing various emotions and scenarios provides a measurable variable that has real-life implications. Our study might provide better ecological validity, as children completed the eye-tracking task with parental assistance at home, compared to the studies conducted in a laboratory. The eye-tracking platform is a reliable measure of gaze fixation time, having been used in multiple other studies [47], and can be performed on almost any computer with a webcam. This means that if this eye-tracking method is deemed to be reliable in screening for ED or its related disorders, it can be employed as a screening tool in communities with relative ease. Secondly, while we used parental reports to measure ED and social function, the measurement tools used, particularly the CBCL and the SRS-2, are valid and reliable and have been used in multiple studies, often used by clinicians to screen for ED-related issues or disorders [61, 62]. Many studies regarding ED in young children only utilise such parental reports, however, our study contributes to the literature by combining these measures with the objective eye-tracking test. Thirdly, the online nature of the study offered some advantages. We were able to reach a larger and more diverse population than we likely would have with an in-person format, thus improving the external validity of our results [63, 64]. Finally, our study utilised a relatively young age group, with children as young as 3 years of age completing the eye-tracking. This demonstrates that even young children can participate in eye-tracking studies, widening the age range that can be explored in studies involving visual attention.

Despite the strengths of this study, it should be noted that the sample size of 50 was smaller than the sample calculated for a moderate effect size of 0.2. Furthermore, as this was only a proof-of-concept study with a small sample size, we did not perform conservative multi-testing correction due to the risk of inflating type II errors; this should be noted when considering the correlations reported. Having procured these significant findings with this modest sample size, further investigation into the correlation between visual attention and ED severity using a larger sample is warranted. Another limitation of this study was the use of only parental report measures to assess ED and social function. Although these measures are reliable in detecting emotional and behavioural problems in children, parents may sometimes over-report or under-report a child's emotional or behavioural problems [44, 45, 61, 65–67]. However, we believe that due to the anonymous online nature of the study, many parents may have felt inclined to be honest about their child’s behaviour, thereby providing a more accurate measure of the behavioural issues. Notwithstanding this, future research should consider using objective measures of ED, such as respiratory sinus arrhythmia or electrodermal activity [68]. Furthermore, our research only screened for the severity of behavioural and emotional issues in the general population, meaning there were no confirmed ED-related diagnoses, such as ASD or ADHD, for us to measure and compare the gaze patterns of. Thus, it is possible that some disorders may have unique attention biases [69].

In summary, the current findings hold the key to a better understanding of cognitive mechanisms underlying how a child manages unpleasant stimuli and pave the way for early screening for emotional problems. In future studies, the use of clinical samples should also be considered, to determine if there are unique attention profiles in different clinical diagnoses.

## Supplementary Information

Below is the link to the electronic supplementary material.

Supplementary file1 (PDF 124 KB)

## Data Availability

The de-identified data underlying this research will be made available to researchers upon reasonable request. Access will be granted to those with appropriate institutional approval and who are compliant with all relevant ethical guidelines. Interested parties should contact Dr. Daniel Lin at pingi.lin@gmail.com to request access. Data will be provided in a format that ensures participant confidentiality is maintained.
