# Supplementary material for: Evaluating the Link between Visual Attention Bias and Emotion Dysregulation of Young Children
Source: Psychiatr Q. 2024 Aug 28;95(4):543–60. doi: 10.1007/s11126-024-10089-4 (PMC11568008; doi:10.1007/s11126-024-10089-4)

Journal name: Psychiatric Quarterly

Title: Evaluating the link between visual attention bias and emotion dysregulation of young children

Submitted: 3rd of April 2024

Supplementary Information

Appendix S1: *The emotional images and their AOIs (delineated by rectangles) that were preloaded into the eye-tracking test*

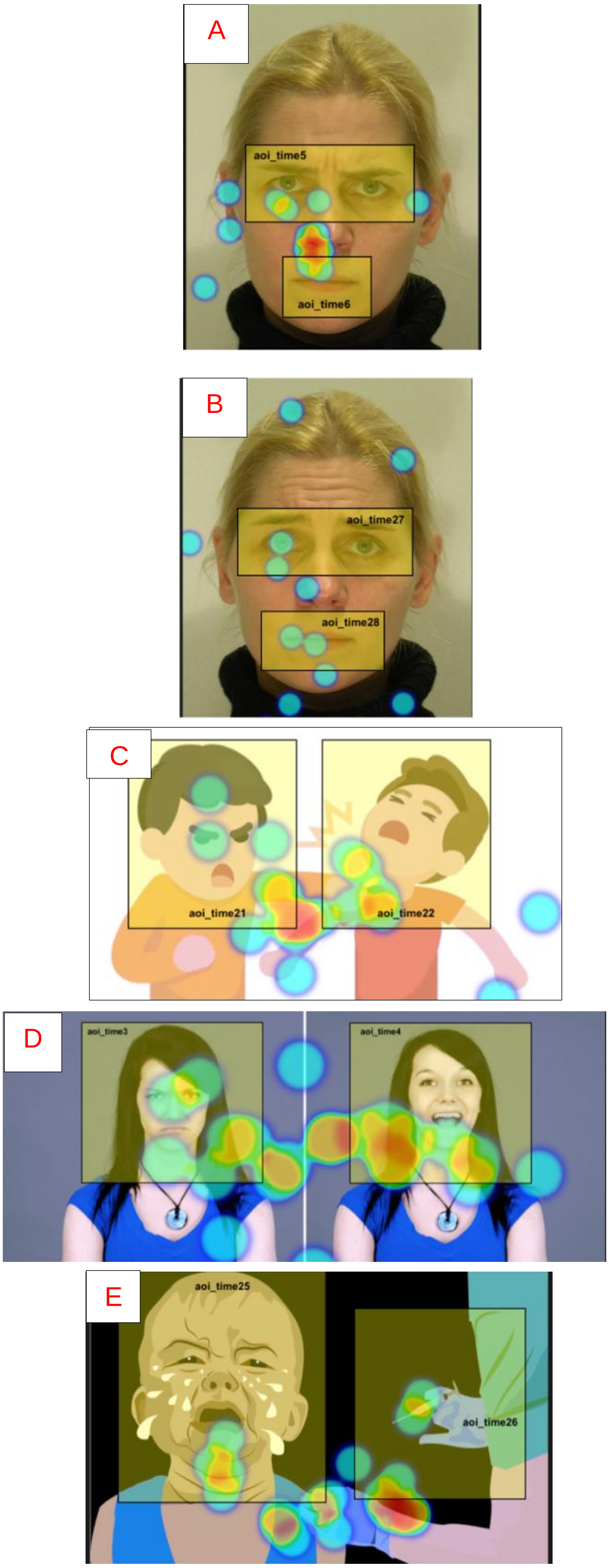

Supplement: Supplementary file 1 — Supplementary file1 (PDF 124 KB) [file 11126_2024_10089_MOESM1_ESM.pdf]
